# Use of Liver Stiffness Measurement for Liver Resection Surgery: Correlation with Indocyanine Green Clearance Testing and Post-Operative Outcome

**DOI:** 10.1371/journal.pone.0072306

**Published:** 2013-08-28

**Authors:** James Fung, Ronnie T. P. Poon, Wan-Ching Yu, See-Ching Chan, Albert C. Y. Chan, Kenneth S. H. Chok, Tan-To Cheung, Wai-Kay Seto, Chung-Mau Lo, Ching-Lung Lai, Man-Fung Yuen

**Affiliations:** 1 Department of Medicine, The University of Hong Kong, Hong Kong, China; 2 Department of Surgery, The University of Hong Kong, Hong Kong, China; The University of Hong Kong, Hong Kong

## Abstract

**Background:**

Liver stiffness measurement (LSM) using transient elastography has recently become available for the assessment of liver fibrosis. Whether LSM can predict the functional liver reserve in patients undergoing liver resection is not certain.

**Aim:**

To correlate liver stiffness measurement (LSM) with indocyanine green (ICG) clearance test and liver biochemistry, and to determine its usefulness in predicting postoperative outcomes in patients undergoing liver resection.

**Patients and Methods:**

Transient elastography and ICG clearance test were performed pre-operatively in 44 patients with hepatocellular carcinoma. The LSM and ICG retention rate at 15 minutes (R15) were correlated with pre-operative factors and post-operative outcomes.

**Results:**

There was significant correlation between ICG R15 and LSM. In patients with LSM ≥11 kPa vs <11 kPa, there was significantly higher ICG R15 (17.1% vs 10.0% respectively, p = 0.025). For patients with ICG R15≥10% compared to those <10%, there was significantly higher LSM (12.0 vs 7.6 kPa respectively, p = 0.015). Twenty-eight patients proceeded to resection. There was a significant correlation between LSM and the peak INR after liver resection (r = 0.426, p = 0.024). There was a significant correlation between ICG R15 and the post-operative peak AST level (r = −0.414, p = 0.029) and peak ALT level (r = −0.568, p = 0.002). The operative time was a significant independent factor associated with post-operative complications and peak INR.

**Conclusion:**

LSM correlated well with ICG R15 in patients undergoing liver resection, and predicted early post-operative complications. Addition of LSM to ICG R15 testing may provide better prognostic information for patients undergoing resection.

## Background

Hepatocellular carcinoma (HCC) is the most common primary tumour of the liver, and the fifth commonest malignancy worldwide [1]. Under normal circumstances, hepatic resection is well tolerated as the liver regenerates rapidly. However, in patients with HCC, the remnant liver is often abnormal due to underlying chronic liver disease. For patients with HCC, bridging fibrosis or established cirrhosis is frequently observed in the non-tumour tissue. It is therefore of paramount importance that the underlying liver function is not severely compromised to cause decompensation and death after liver resection. Stringent pre-operative assessment is therefore essential to prevent post- resection morbidity and mortality. Routine liver biochemistry, the Child Pugh classification, and the Model for End-stage Liver Disease (MELD) score provides an estimation of the severity of liver disease, but cannot predict the liver synthetic function reserve, or the ability of the liver to regenerate after resection [2], [3], [4].

Indocyanine green (ICG) clearance test, a more objective measurement for liver reserve is commonly adopted in many centers because of its high predictive value for postoperative outcome after liver resection [5]. ICG is a synthetic dye which binds completely to albumin and β-lipoprotein and is eliminated by the liver into the bile virtually unchanged without any extrahepatic metabolism or excretion [6]. Excretion of ICG is dependent on hepatic adenosine triphosphate concentration, and decreased levels may reflect reduced ability to regenerate after liver resection. The ICG retention value at 15 minutes (ICG R15) after injection is approximately 10% in normal persons. A cut-off value for a safe major hepatectomy is 14%, although the cut-off may be higher for centres with more operative experience, patients with adequate remnant liver volume and/or with limited resections [7], [8]. Infrared digital measurement based on pulse spectrophotometry can be used at bedside to replace repetitive blood taking, and has been shown to be accurate in determining blood ICG concentration [9], [10], [11].

Recently, liver stiffness measurement (LSM) by transient elastography has become available for the non-invasive assessment of liver fibrosis. LSM has been shown to correlate well with liver biochemistry, liver histology, and predictive of long term outcome in Asian patients with chronic hepatitis B [12], [13], [14]. In patients with established cirrhosis, higher LSM may also predict complications of cirrhosis including the presence of varices and the development of HCC [15], [16]. A pilot study also demonstrated that LSM may be useful in predicting development of postoperative hepatic insufficiency after resection for HCC [17]. Therefore, LSM may be able to predict the functional liver reserve indirectly through the assessment of fibrosis level.

The current study aims to correlate LSM with ICG clearance test and routine liver biochemistry, and to determine its usefulness in predicting postoperative outcomes in patients with HCC undergoing liver resection.

## Patients and Methods

Patients with HCC scheduled for potential liver resection from October 2010 to December 2011 at Queen Mary Hospital, Hong Kong, were prospectively recruited. All patients enrolled in the study underwent ICG clearance testing (Pulsion Medical Systems AG, Munich, Germany) and LSM using transient elastography (Echosens, Paris, France) on the same day. Routine liver biochemistry was performed. Basic patient demographics and operation details were recorded. Pre- and post-operative laboratory results and outcome were monitored. Written informed consent was obtained from all subjects. This study was approved by the Ethics Committee/Institutional Review Board of the University of Hong Kong and Hospital Authority Western Cluster. All clinical investigations were conducted according to the principles expressed in the Declaration of Helsinki.

### ICG Clearance Test

The ICG clearance test was performed with the patient fasted for 6 hours. ICG was administered intravenously at a dose of 0.5 mg/kg. The ICG Plasma Disappearance Rate (PDR) was measured transcutaneously using a near-infrared finger clip sensor. The ICG retention rate at 15 minutes (R15) was then calculated. The ICG retention value at 15 minutes (ICG R15) after injection is approximately 10% in normal persons, and this value was used for stratification of patients in the current study [18].

### Liver Stiffness Measurement

This procedure has been well described previously [12]. The LSM was performed by a single experienced operator (who had performed over 2000 scans). The operator was blinded of the ICG results of the patients. At least 10 valid measurements were obtained for each patient. The median values of the validated measurements for each patient were representative of the liver stiffness and expressed in units of kilopascals (kPa). The results were valid only for the final analysis if the success rate was greater than 50% and the interquartile range (IQR)-to-liver stiffness ratio was below 30%. A cut-off value of 11 kPa has been shown to be predictive of the presence of cirrhosis, and this was used for stratification in the current study [19].

### Statistical Analyses

All statistical analyses were performed using SPSS version 17.0 (SPSS Inc, Chicago, IL). Chi-squared test or Fisher’s exact test was used for categorical variables when appropriate. Continuous variables with skewed distribution were analyzed using Mann-Whitney test. The comparison of different median values of more than two groups was made by Kruskal-Wallis test. The correlation co-efficient was calculated using Spearman test. Multivariate analysis was performed using linear regression. The area under receiver operating characteristic (AUROC) curve was used to derive an optimal cut-off of liver stiffness measurement for predicting short-term complications after liver resection. A p-value of<0.05 was considered statistically significant.

## Results

**Figure 1:**
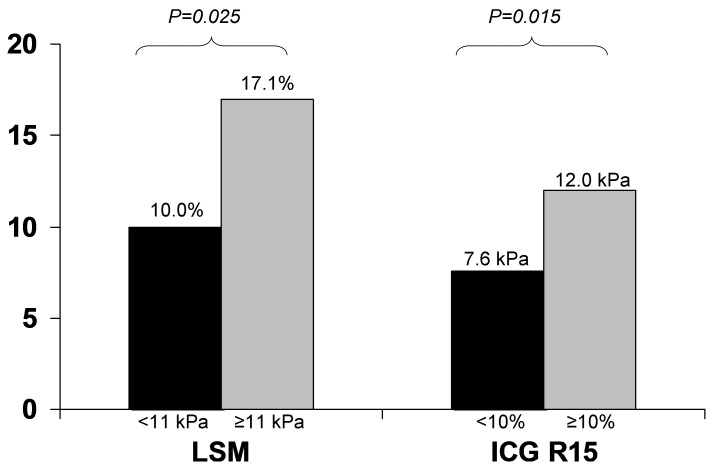
Comparison between LSM and ICG R15.

A total of 68 patients were enrolled during the recruitment period in the current study. Twenty four were excluded from the final analysis due to suboptimal (22 patients had high IQR/LSM ratio) or invalid LSM (2 patients had less than 10 valid measurements). Forty-four patients were included in the final analysis. Thirty six (82%) were male. The median age was 59 years (range, 46–75). The basic patient demographics and laboratory data are summarized in table 1. The correlation between ICG clearance and LSM with age and other routine laboratory parameters are summarized in table 2. There were significant correlations between ICG R15 and LSM, bilirubin, albumin, international normalized ratio (INR), platelet levels, and MELD scores, and between LSM and AST, albumin, INR, and MELD scores. A previous study of CHB patients identified a LSM cut-off value of 11 kPa for the presence of cirrhosis [19]. In our patients with LSM ≥11 kPa vs <11 kPa, there was significantly higher ICG R15 (17.1% vs 10.0% respectively, p = 0.025), lower albumin (38 vs 45 g/L respectively, p<0.001), higher INR (1.1 vs 1.0 respectively, p<0.001), and lower platelet counts (131 vs 170×10^9^/L respectively, p = 0.043). The normal ICG R15 is <10% [18]. For patients with ICG R15≥10% compared to those <10%, there was significantly higher LSM (12.0 vs 7.6 kPa respectively, p = 0.015), higher bilirubin (12 vs 8 umol/L respectively, p = 0.039), lower albumin (41 vs 46 g/L respectively, p<0.001), higher INR (1.1 vs 1.0 respectively, p<0.001), and lower platelet counts (132 vs 178×10^9^/L respectively, p = 0.027). The comparison between LSM and ICG R15 is shown in figure 1. Using multivariate analysis, only albumin remained a significant factor associated with both ICG R15 and LSM (p = 0.011 and p = 0.007 respectively).

**Table 1:** Basic patient demographics and laboratory data.

Parameter	Value
Total patients (N)	68
Invalid fibroscan	24
Numbers in final analysis (n)	44
Male sex, n(%)	36 (82%)
Age (years)	59 (46–75)
Bilirubin (umol/L)	9 (5–29)
ALT (U/L)	39 (16–167)
AST (U/L)	47 (21–263)
Albumin (g/L)	42 (26–52)
Creatinine (umol/L)	80 (57–485)
INR	1.0 (0.9–1.2)
AFP (ng/ml)	37 (3–539700)
Plalelet (x10^9^/L)	155 (23–466)
MELD score	7.3 (6.4–13.7)
HCC size (cm)	3.5 (1–14)
Liver stiffness (kPa)	10.2 (3.3–75.0)
ICG R15 (%)	11.8 (5.9–40.7)

Continuous variables are expressed as median values (range).

**Table 2:** Correlation between LSM, ICG R15 and other laboratory parameters.

Parameters	Liver stiffness r co-efficient	Liver stiffness P value	ICG R15 r co-efficient	ICG R15 P value
Liver stiffness	–	–	0.342	0.023
ICG R15	0.342	0.023	–	–
Age	−0.032	0.836	0.100	0.517
Bilirubin	0.136	0.378	0.349	**0.020**
ALT	0.228	0.137	−.080	0.605
AST	0.455	**0.002**	0.095	0.541
Albumin	−.0710	**<0.001**	−0.525	**<0.001**
Creatinine	0.071	0.649	0.141	0.361
INR	0.682	**<0.001**	0.548	**<0.001**
AFP	0.140	0.369	0.081	0.605
Platelet count	−0.176	0.253	−0.322	0.033
MELD score	0.431	**0.003**	0.519	**<0.001**
HCC size	0.248	0.108	−0.211	0.174

### Post-operative Outcomes

Of the 44 patients with valid LSM, 28 proceeded to liver resection. The remaining 16 patients underwent one of the following treatments: loco-ablative therapy, chemotherapy, or palliation. Of the 28 patients who underwent resection, 10 had segmentectomy, 4 had sectionectomy, 5 had wedge resection, and 9 had lobectomy. The characteristics of the resected patients are shown in table 3. The correlations between operative and post-operative parameters with pre-operative parameters are shown in table 4. There was significant correlation between operative blood loss and HCC size (r = 0.632, p<0.001) and operative time (r = 0.510, p = 0.006), and between operative time and HCC size (r = 0.573, p = 0.001).

**Table 3:** Characteristics of patients undergoing liver resection.

Parameters	Value
Number of resected patients	28
Presence of cirrhosis	19 (68%)
Child Pugh Class (A:B:C)	27∶ 1∶0
MELD score	7.2 (6.4–11.7)
Number of tumours	1 (1–6)
Size of largest HCC (cm)	4.2 (2–14)
Hepatitis B	23 (82%)
Hepatitis C	2 (7%)
Cryotpgenic cirrhosis	2 (7%)
Gastrointestinal stromal tumour	1 (4%)
Segmentectomy	18
Sectionectomy	4
Wedge resection	5
Lobectomy	9
Operation time (minutes)	343 (45–1149)
Blood loss (mls)	517 (50–2000)
Bilirubin (umol/L)	33 (18–79)
AST (U/L)	344 (66–3000)
ALT (U/L)	312 (39–3000)
INR	1.4 (1–2.3)
Length of hospital stay (days)	6 (3–21)

Continuous variables are expressed as median values (range).

**Table 4:** Correlation between pre- and post- operative parameters.

Parameters	Peak Br	P value	Peak ALT	P value	Peak AST	P value	Peak INR	P value	Hospital Stay	P value
Pre-operative										
Age	−0.200	0.308	−0.116	0.556	−0.091	0.646	−0.087	0.659	0.037	0.851
MELD score	0.165	0.402	−0.252	0.196	−0.219	0.264	0.126	0.524	−0.029	0.882
AFP	0.447	**0.019**	−0.161	0.423	0.033	0.871	−0.064	0.753	−0.179	0.372
HCC size	0.176	0.369	0.359	0.060	0.369	0.053	0.605	**0.001**	0.615	**0.001**
ICG R15	−0.015	0.939	−0.568	**0.002**	−0.414	**0.029**	−0.169	0.390	−0.048	0.808
LSM	0.274	0.158	−0.272	0.161	−0.132	0.504	0.426	**0.024**	0.185	0.345
Operative										
Operation time	0.026	0.895	0.505	**0.006**	0.506	**0.006**	0.768	**<0.001**	0.619	**<0.001**
Blood loss	0.169	0.391	0.261	0.179	0.334	0.082	0.659	**<0.001**	0.412	**0.029**

### Correlation with LSM

**Figure 2:**
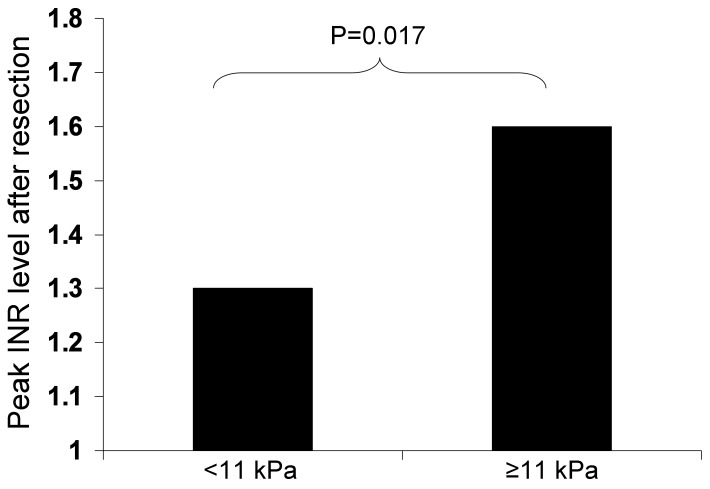
Pre-operative liver stiffness associated with higher post-resection INR.

There was a significant correlation between LSM and the peak INR after liver resection (r = 0.426, p = 0.024). No significant correlation was observed between LSM and peak levels of bilirubin, AST, and ALT, and length of hospital stay after resection (table 4). In patients with LSM >11.0 kPa, a larger HCC, greater blood loss, and a higher peak INR after resection was observed. The comparison between pre-operative LSM and post-resection INR is shown in figure 2. No significant difference was observed with respect to operative time, peak levels of bilirubin, AST, and ALT after resection, and length of hospital stay.

Apart from LSM, other parameters significantly correlated with peak post-operative INR included pre-op AST (r = 0.424, p = 0.025), HCC size (r = 0.605, p<0.001), and operative time (r = 0.768, p<0.001). Using multivariate analysis, only the operative time remained significantly associated with the peak INR.

### Correlation with ICG R15

**Figure 3:**
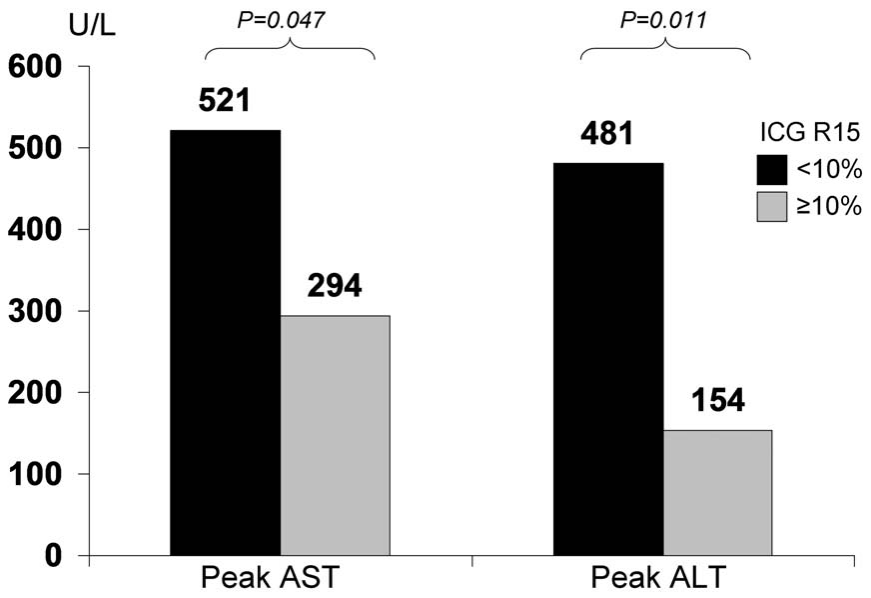
Pre-operative ICG R15 associated with higher post-operative transaminases.

There was a significant correlation between ICG R15 and the post-operative peak AST level (r = −0.414, p = 0.029) and peak ALT level (r = −0.568, p = 0.002). There was no correlation between ICG R15 and post-operative peak bilirubin and INR levels, and length of hospital stay (table 4). In patients with ICG R15≥10%, vs <10%, there was a significant lower peak levels of AST (294 vs 521 U/L respectively, p = 0.047) and ALT (154 vs 481 U/L respectively, p = 0.011) after resection (as shown in figure 3). There was no significant difference with HCC size, amount of operative blood loss, length of operative time, length of hospital stay, and peak level of bilirubin and INR.

In addition to ICG R15, other factors that was significantly correlated with peak AST included pre-operative platelet levels (r = 0.379, p = 0.047) and operative time (r = 0.506, p = 0.006). For peak ALT, other factors with significant correlations included pre-operative bilirubin (r = −0.419, p = 0.027), pre-operative albumin (0.479, p = 0.010), pre-operative INR (r = −0.426, p = 0.024), pre-operative platelet count (r−0.412, p = 0.030), and operative time (r = 0.505, p = 0.006). Using multivariate analysis, only the operative time remains significantly associated with both peak AST and ALT levels after resection.

Of the 28 patients, 9 had hepatic inflow occlusion intra-operatively, and there was no significant differences when compared to the 19 patients without occlusion with respect to peak AST (437 vs 284 U/L respectively, p = 0.156) and ALT (344 vs 154 U/L respectively, p = 0.085).

### Presence of Cirrhosis

In patients who underwent liver resection, the surrounding non-tumour tissue was assessed histologically for the presence of cirrhosis. Of the 28 patients, 19 had evidence of established cirrhosis on histology. In cirrhotic patients versus non-cirrhotic patients, there was significantly higher LSM (9.9 vs 6.3 kPa respectively, p = 0.012). There was no difference between cirrhotic patients vs non-cirrhotic patients with respect to ICG R15 (14.6 vs 10.0% respectively, p = 0.498). There were no significant differences between cirrhotic and non-cirrhotic patients with respect to the operative time, amount of blood loss, and peak levels of post-operative bilirubin, AST, ALT, and INR.

### Short-term Outcome

Of the 28 patients undergoing resection, all were discharged well with a median hospital stay of 6 days (range, 3–21). We defined post-operative complications as any deviation from the normal recovery course with or without the need for pharmacological treatment or surgical, endoscopic, and radiological interventions. Seven patients had evidence of early (<1 month) post-operative complications, with a median time from operation to development of complication of 11 days (range, 2–17). Ascites developed in 4 patients and was the most common complication. The remaining 3 complications included a combination of wound leakage and pneumonia. The only factor significantly associated with the development of complications was the operative time (p = 0.008). Age, ICG, LSM, albumin, platelet, MELD score, the presence of cirrhosis, HCC size and operative blood loss were not significantly associated with the development of complications.The HCC size, LSM, intra-operative blood loss, and operative time were significantly associated with the post-operative peak INR. After multivariate analysis, only the operative time remained the only significant factor (p = 0.002), whereas ICG, LSM, albumin, platelet count, MELD score, presence of cirrhosis, HCC size and intra-operative blood loss were not significant factors.

## Discussion

For patients undergoing liver resection, assessment of the liver function is essential to ensure that sufficient liver function remains after resection to allow for regeneration of the remnant liver and to prevent decompensation and death. Current methods to assess liver function include using the Child Pugh score, MELD score, and the ICG R15. Routine laboratory, endoscopic and radiological assessment can also be used to determine the presence of underlying portal hypertension. More recently, transient elastography has become available as a non-invasive method for assessing liver fibrosis and cirrhosis. Previous studies have shown good correlation between LSM and the degree of fibrosis [12], [13], [14]. In patients with established cirrhosis, higher LSM was also correlated with complications of cirrhosis, including the presence of varices [13], [15], [16].

In contrast to LSM, ICG testing determines the functional capacity rather than the degree of architectural change in the liver. Although ICG is extracted exclusively by the liver with negligible extrahepatic elimination, the clearance rate is dependent on hepatic blood flow and plasma volume. Therefore, it remains an imperfect test for assessing hepatic functional reserve. A previous pilot study has suggested a potential role for LSM to predict post-resection hepatic insufficiency [17]. A more recent study demonstrated that elevated LSM was an independent predictor of postoperative liver failure; those with LSM <14.8 kPa had no liver failure [20]. Another study showed that LSM >12.0 kPa predicted worst outcomes after hepatectomy, and was better than ICG test in predicting major postoperative complications [21].

In the current study, significant correlation between ICG R15 and LSM was demonstrated in patients undergoing liver resection. However, after multivariate analysis, only albumin remained a significant factor that was correlated with either ICG R15 or LSM. This would suggest that the correlation between ICG R15 and LSM is not independent of other more important factors such as the level of serum albumin. This is not surprising given that ICG R15 is a functional test whereas LSM is a structural assessment. In fact, in patients with cirrhosis, the LSM was significantly higher than non-cirrhotic patients, in comparison to ICG R15 where no differences were observed.

There was also significant correlation of LSM and ICG R15 with post-operative outcomes. Interestingly, the post-operative peak INR was correlated with LSM whereas the post-operative peak AST and ALT were inversely correlated with ICG R15. The higher INR observed is consistent with the fact that those patients with higher LSM have more advanced fibrosis/cirrhosis, resulting in a higher INR after resection. This implies that the risk of liver decompensation may be higher in patients with high LSM who undergo liver resection. This may be an important consideration for those who have major liver resection. It will be interesting to define in future studies, the role of LSM to stratify post-operative risks in patients with different extent of liver resection.

Elevation of transaminases is commonly observed after liver resection. The causes are multifactorial, including the duration of operation, transaction surface and any ischemic injury to the liver during operation. In the current study, only the length of operation remains significantly associated with the post-resection peak INR and transaminase after multivariate analysis. However, it should be noted that the results of the multivariate analysis may be affected by the relatively small number of patients (n = 28) who eventually underwent liver resection. Further studies are required to examine specifically whether LSM and ICG R15 are important measurement to correlate liver decompensation and hepatocyte damage respectively after liver resection.

There are several limitations to the current study. Firstly, there was an unusually high rate of invalid FS (35%), so the number included in the final analysis was reduced. This is most likely due to the higher IQR that is often observed in patients with established cirrhosis. In fact, only 2 of the patients excluded because of invalid LSM were due to insufficient success rate. Secondly, the patients included in the study have already been selected as potential candidate for resection based on their normal clinical parameters and the absence of overt portal hypertension. To determine a cut-off value of ICG R15 and LSM whereby resection is contra-indicated would require a proportion of patients who decompensated after resection. As all 28 patients were discharged without evidence of severe decompensation, this would suggest that at least in pre-selected patients with compensated cirrhosis and without evidence of portal hypertension, the addition of LSM may not provide further decisional value in addition to ICG R15. However, it is noteworthy that the majority of patients underwent minor resection rather than lobectomy. LSM may still have a role in assessing the risk of decompensation in patients undergoing major hepatectomy and this needs a further study to clarify.

In conclusion, LSM correlated well with ICG R15 in patients undergoing liver resection, and may predict complications in the early post-operative phase. Further studies are required to determine whether the addition of LSM to ICG R15 testing has any added benefit in the pre-operative assessment to select out patients suitable for hepatectomy.
